# A Multi-Residue Analytical Method for Assessing the Effects of Stacking Treatment on Antimicrobial and Coccidiostat Degradation in Broiler Litter

**DOI:** 10.3390/ph17020203

**Published:** 2024-02-04

**Authors:** Solomon Efriem, Malka Britzi, Stefan Soback, Chris Sabastian, Sameer J. Mabjeesh

**Affiliations:** 1 The Robert H. Smith Faculty of Agriculture, Food and Environment, Hebrew University, P.O. Box 12, Rehovot 7610001, Israel; solomone@moag.gov.il (S.E.); chriss@savion.huji.ac.il (C.S.); 2 National Residue Control Laboratory, Kimron Veterinary Institute, Beit Dagan 5025001, Israel; malkab@moag.gov.il (M.B.); stefan.sobock@gmail.com (S.S.)

**Keywords:** analytical method, antimicrobials, coccidiostats, LC/MS/MS, multi-residue analysis, broiler litter

## Abstract

Antimicrobial drugs and coccidiostat compounds are commonly used in poultry farming. These compounds are subsequently excreted and released into the environment via broiler litter (BL) and can re-enter the food chain as fertilizer or animal feed. Such residue in animal feed can encourage the appearance of antibiotic-resistant bacteria as well as toxicity. Most analytical methods used to identify and quantitate these drug residues are traditional, and are specific to some antimicrobials and present limitations in assessing complex matrixes like BL. The aim of this study was to develop a multi-residue analytic method for assessing 30 antimicrobial drugs and coccidiostats associated with BL. We investigated the presence and the effects of biotic stack treatment on the degradation of drug residue in BL. Liquid-liquid extraction (LLE) and solid phase extraction (SPE) were replaced by Quick, Easy, Cheap, Effective, Rugged, and Safe (QuEChERS) clean-up steps and detected by liquid chromatography mass spectrometry (LC/MS/MS). Results show that a wide spectrum of residues were detected from 0.4 to 8.9 mg kg^−1^. Following lab-scale stacking treatment, tilmicosin and eight coccidiostats persisted in BL (26–100%). This research supports the need for better understanding, regulation, and management of the use of BL that might carry a high risk of residue drugs.

## 1. Introduction

Global poultry meat consumption has been growing rapidly during the last two decades for various reasons, including the desire for healthier foods and calls for inexpensive sources of protein. Accordingly, a total of 280 million broilers were produced in Israel during 2021 via the implementation of intensive production methods involving reduced energy consumption and a 40-day-growth period [1]. During the broiler growth cycle, 0.3–0.85 kg of litter is produced per animal. In 2021, 336,000 tons of broiler litter (BL) were produced and used for agricultural soil fertilization, soil amendments and, in the form of dried broiler litter (DBL), as ruminant feed [2,3,4]. Broiler litter contains more crude protein and less ash, as compared to layer hen or turkey litter. 

Of the coccidiostats and antimicrobials utilized in poultry farming, 70–80% are excreted and released into the environment [5,6]. Coccidiostats (ionophores, nicarbazine, and clopidol) are compounds used in poultry farming for prophylactic or growth promotion purposes. In addition to coccidiostats, groups of therapeutic antimicrobial agents are also used for treatment and prevention of infection. According to the FDA, despite a decreasing trend, 6.1 million kg of antimicrobials and 4.2 million kg of ionophores were sold to US animal producers in 2019, of which 3% of the antimicrobials and 25% of the ionophores were intended for use with poultry [7]. Thus, feeding animals with DBL involves the risk of spreading bacteria, antimicrobial resistance, and toxins, as well as drug residues that can cause potential public health hazards, in addition to morbidity, and even mortality in animals [8,9,10]. 

The most widely used antimicrobial and decomposition treatments of BL used as animal feed are aerobic (i.e., forced aeration), anaerobic (i.e., ensiling), and stacking. Stacking treatment is a semi-aerobic treatment involving passive pile windrows and in which turning is not required for aeration, as in compost. Efficiency of decomposition depends on litter composition, the litter environment, physical properties of the litter, and the quality of the bedding and feed composition. Environmental conditions include moisture, aeration, pore space, and flow of water for microbial activity, while physical factors include location (i.e., surface placement), insulated treatment space, litter volume and freshness, windrow height and width, and particle size. Litter hydrophobicity and the carbon/nitrogen ratio (20–30:1) also affects the ability to decompose litter quickly and completely. Specifically, a low carbon/nitrogen ratio reduces microbial activity and causes reduced decomposition, whereas high ratios also slow the decomposition process. The persistence and degradation of certain antimicrobials and ionophores in BL have been studied in terms of composting, aerobic digestion, and the impact of soil and aquatic environments [11,12,13,14,15,16]. However, the effect of stacking BL treatment on the degradation of pharmaceutical compounds is still largely unknown [17,18,19]. 

Recycling BL in Israel and worldwide started in 1940. Dry BL is used as animal feed due to economic and environmental considerations. Broiler litter is also used as emergency feeding when not enough sources of ruminant feed exist. The three major dried BL producers/suppliers in Israel are focused on compost in the north-west (stacking treatment), south industries (aerobic treatment), and north-east antimicrobial independent farms (aerobic treatment). Jointly, they supply 70,000–100,000 tons of dried BL. The remaining 19% of BL used as animal feed undergoes ensiling treatment by farmers or is utilized without any treatment. Pasture-based cattle are the most BL using agents, with 48% used for agriculture following either composting treatment or as is.

The challenge in determining antimicrobial and coccidiostat residues in BL is related to their physicochemical properties, as well as components of the BL matrix that include moisture, organic, and inorganic matter. Multiple antimicrobials and coccidiostats have been identified by LC/MS/MS in matrices other than BL [20,21]. However, traditional analytical methods for characterizing BL remain limited. Cleaning procedures based on solid phase extraction columns are specific to some antimicrobials, with sample preparation time being long and high amounts of solvents used. At the same time, fast multi-analyte detection methods using QuEChERS have been developed for use with food, vegetables, fruits, urine [22,23,24,25], and swine manure (not including polar antimicrobials and coccidiostat drugs [26].

Liquid-liquid extraction used in previous studies has been shown to be an effective method of separating compounds in two immiscible liquids (aqueous and organic solvents). The problems with this method include the emulsion process in which the adsorption of analytes to other matrix molecules, e.g., protein, occurs. Additionally, the natural solubility of the two phases causes emulsion in the extraction phase of residue analytical methods. To overcome this emulsion, the addition of salts, heating or cooling, filtration, other organic solvents, and centrifugation were used. Yet, these methods were not always effective and extraction times were lengthy [27,28,29,30,31,32,33]. In a previous study, errors during chromatographic analysis were broken down to 30% from the sample processing, 19% by the analyzer (operator), and 11% by the choice of column used [34]. In traditional methods, increased sample preparation time reduces the recovery rate and leads to a loss of analytes and high Relative Standard Deviations in repeatability and reproducibility. According to Majors’ data from 2013 [34], 61% of time spent on typical chromatographic analysis was for sample preparation.

In the present study, we report the first multi-residue analysis method for containing five different antimicrobial classes, and both ionophores and synthetic coccidiostats (together comprising 30 pharmaceutical compounds at the residue level). The method was used to investigate the presence, persistence, degradation, toxicity concentration of residues, and the relation between residue drugs and resistant bacteria in BL used as ruminant feed. The target sensitivity of the method was set to closely correlate with the existing maximum residue limits (MRL) for these compounds in poultry liver. This was considered essential for the purpose of evaluating the potential for building resistant bacterial populations in the litter and the environmental spread thereof. Our multi-residue analytical method as shown in this study is characterized by low interference, good recovery, and selectivity for antimicrobial and coccidiostat residues in BL, reduces sample preparation steps, and reduces the high analytical cost. 

This research supports the need for better understanding, regulation, management, and proper use of animal waste so as to protect and reduce the numbers of antibiotic-resistant bacteria and the levels of drug residues that carry a high risk for animals, humans, and the environment. This study is of interest to agriculture, public health, environment pollution, analytical chemistry, and waste management researchers.

## 2. Results

### 2.1. Method Validation

#### 2.1.1. Suitability (Repeatability)

The results for coccidiostats and tetracycline, sulfonamide, fluoroquinolone, macrolide, and beta-lactam groups of antimicrobials had a relative standard deviation (RSD) < 20%, except for oxytetracycline at high concentrations (Table 1). Medium level recoveries at 500 µg kg^−1^ were 80–120% and RSD < 20% (shown in the Table S2).

#### 2.1.2. Inter-Day Recovery (Reproducibility)

The results for coccidiostats and sulfonamide, fluoroquinolone, macrolide, and beta-lactam groups of anti-microbials had an RSD <20%, except for norfloxacin and chlortetracycline (Table S3).

#### 2.1.3. Specificity and Selectivity

Good signal-to-noise ratios, selectivity, specificity, and low interference at retention time (RT) except for decoquinat (S/N < 10, Table S6) were observed. The coefficient of variation (CV%) of blank sample areas for sulfachlorpyridazine and erythromycin were 20.7 and 21.8%, respectively (Table S6).

**Figure 1:**
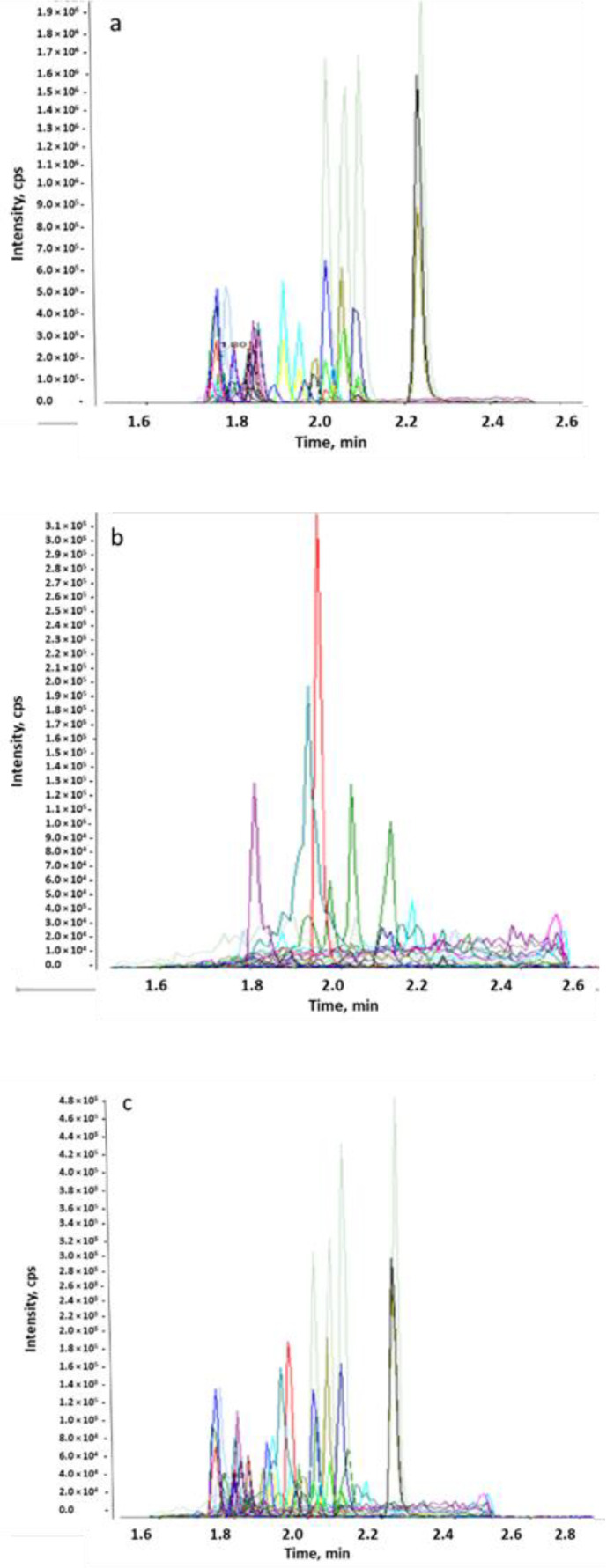
Chromtograms of antimicrobials in standard solution (**a**), blank matrix (**b**), and spiked matrix (**c**).

Representative chromatograms of antimicrobials in standard solution, blank matrix, and spiked matrix are presented in Figure 1. Chromatograms of beta-lactams and coccidiostats are presented in Figure S2, Figure S3 and Figure S4 respectively.

The peaks of a few transitions observed in blank matrix differ in retention time and do not co-elute with the relevant analytes. For all others, the area is lower than 30% of LOQ. 

### 2.2. Identification and Quantification of BL Samples

Broiler litter samples were collected at four different locations. Altogether, 18 antimicrobials and coccidiostats were detected in 42 batches of samples during 2019–2021. Most samples were positive for one or more of the analyzed compounds. Members of the tetracycline, fluoroquinolone, and coccidiostat groups were detected more in the northwest and south of Israel, as compared to the northeast and other locations in the country (Figure 1). Macrolides and beta-lactams were not detected at all. Indeed, beta-lactam antimicrobials are not stable in the solution or in the environment. 

**Figure 2:**
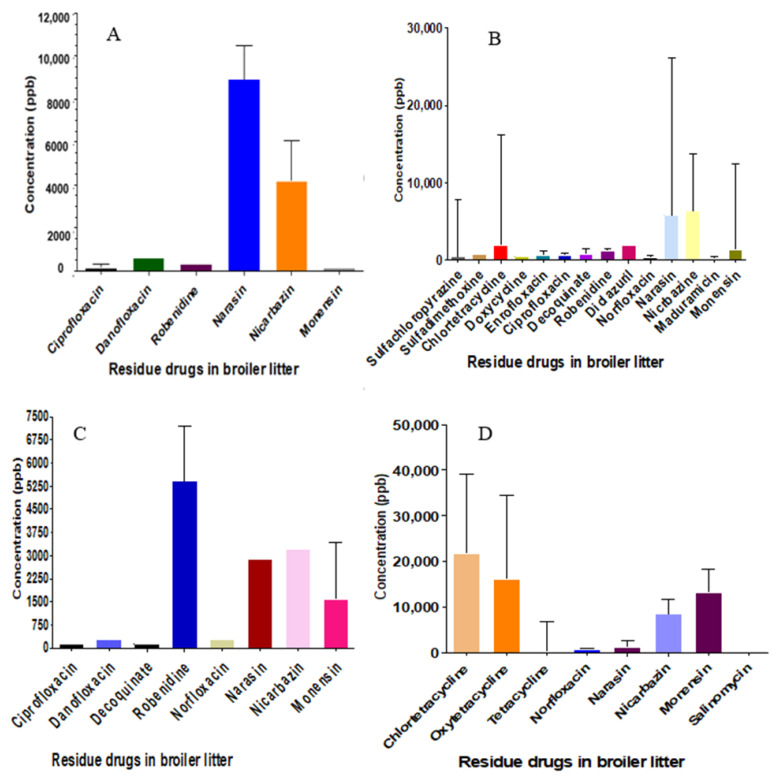
Medium concentration ppb (µg kg^−1^) of antimicrobial and coccidiostats in broiler litter in Israel (2019–2021): (**A**) northeast (*n* = 11), (**B**) northwest (*n* = 11), (**C**) south (*n* = 15) and (**D**) other regions (*n* = 5). Error bars represent the SD.

In Figure 2, we show results for 18 antimicrobial and coccidiostat drugs detected in BL. The prevalence (and medium concentration; mg kg^−1^ (ppm)) of drug residues in BL were: narasin 47% (2.88), nicarbazine 59.5% (6.06), monensin 57% (6.97), robendine 14% (1.35), salinomycin 4.2% (0.17), decoquinate 7.1% (0.19), diclazuril 4.2% (1.92), maduramycin (0.28 mg/kg), sulfachloropyrazine 9.5% (0.46), sulfadimethoxine 4.2% (0.85), norfloxacin 19% (0.7), danofloxacin 4.7% (0.43), enrofloxacin (9.5% (0.68), ciprofloxacin (14.3% (0.22), oxytetracycline 21.4% (8.92), tetracycline 23.8% (0.152), doxycycline 4.2% (0.49), and chlortetracycline 33% (8.92).

### 2.3. Antimicrobial and Coccidiostat Residue Degradation upon Stacking Treatment

**Figure 3:**
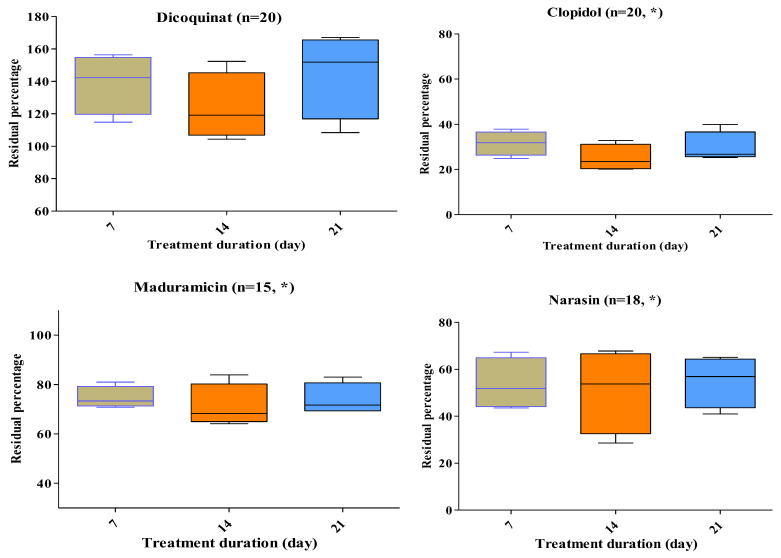

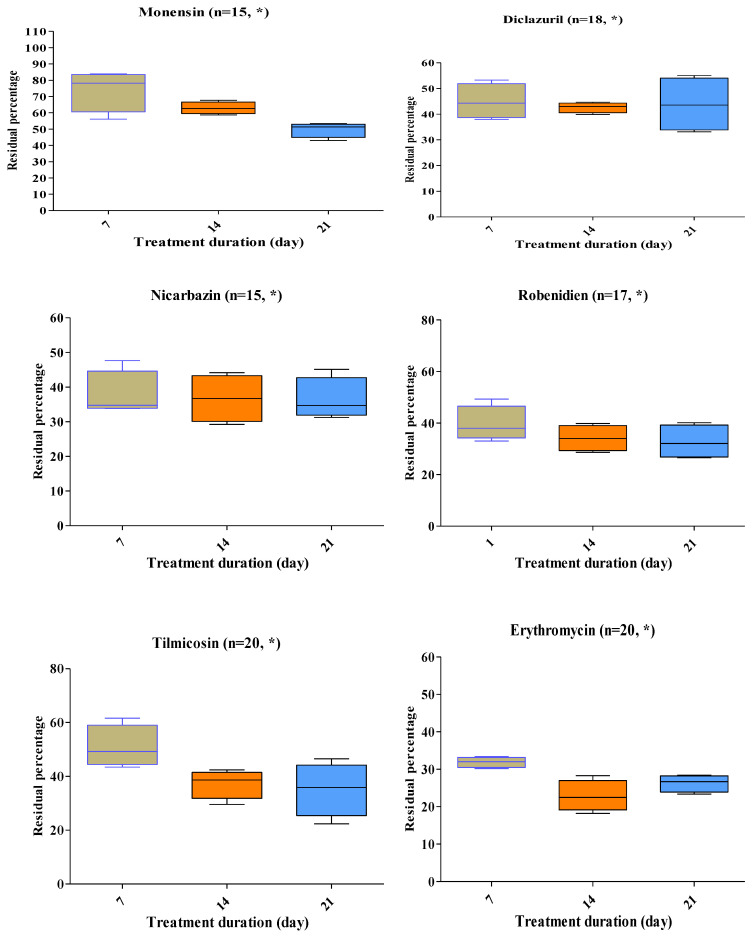
Residual percentage of ten antimicrobial and coccidiostat drugs after stacking broiler litter treatment. * (Significant).

Four representative antimicrobials (tetracycline, ciprofloxacin, sulfisoxazole, and amoxicillin) at high concentrations were degraded >95%, except for erythromycin (74%), by the stacking treatment (Figure 3). All five antimicrobial groups, namely, tetracycline, fluoroquinolones, macrolides, sulphonamides, and beta-lactams (i.e., tetracycline, doxycycline, oxyteteracycline, chlortertracycline, amoxicillin, ciprofloxacin, danofloxacin, enrofloxacin, norfloxacin, sulfisoxazole, sulfachloropyrazine, sulfachloropyridazine, sulfadiazine, sulfadimidine, sulfadoxine, sulfadimethoxine, tylosine, and erythromycin) at low residue concentrations were degraded >95%, except for tilmicosin (65%; Figure 3). Other than salinomycin, all eight coccidiostats persisted in BL (Figure 3). During treatment, the temperature and pH rose to 45–55 °C and 7–8.8, respectively.

Residue percentages (±SEM) after three weeks of treatment at different spiked concentrations of 0.5, 1, 1.5, and 2 ppm of dicoquinat, clopidol, maduramicin, narasin, monensin, diclazuril, nicarbazin, robenidien, tilmicosin, and erythromycin (Figure 3). 

The experimental data showed that high rates of degradation were obtained after seven days of stacking treatment. Tilmicosin persisted more than macrolide groups (erythromycin and tylosin) at low spiked concentrations of 0.5–2 mg kg^−1^ (ppm). Residue percentages (±SEM) upon stacking treatment were 35% (±2.6) for tilmicosin. For clopidol, the value was 36.4% (±3.2). Tilmicosin and clopidol are drugs not often used on Israel broiler farms. The four coccidiostats used in Israel at low frequencies are decoquinat, diclaziuril, maduramycin, and robendien. The residue percentage of these coccidiostats were >100 (±9.9), 43.7 (±5.7), 73, and 32.7% (±3), respectively, after stacking treatment. For the most widely used coccidiostats, namely, narasin, monensin, and nicarbazine, degradation percentages upon stacking treatment were 54.9 (±5.4), 49 (±2.1), and 36.5% (±3), respectively.

## 3. Discussion

The development of simultaneous multi-class drug residue determination in liquid chromatography tandem mass spectrometry (LC/MS/MS) is a challenging task due to low concentration of compounds of interest in a complexed matrix such as BL, that causes a high background of undesired compounds. The real challenge is related to the variety of chemical properties of different classes of drugs that should be extracted simultaneously from such a complicated matrix. The extraction procedure has to be efficient enough to enable good sensitivity as well as selectivity, in order to allow precise identification and quantification of all analytes of interest at the target level of concentration.

Analytical methods previously published and used for detection and quantification of drug residues in animal manure are mostly for sulfonamides, tetracycline, and fluoroquinolones [35] in liquid manure and not in BL. The concentrations of specific antimicrobial residues were also calculated in dry and wet manure samples [35]. Previous studies showed the difficulty in comparing and understanding the real concentration of drug residues in dry and wet manure. 

The wide range of physical, chemical, and biological properties of antimicrobials and coccidiostats, including 54.8–64% natural organic matter, minerals, metals, and moisture [36,37], create a highly challenging matrix for compound extraction. Antimicrobials and coccidiostats in BL present variable chemical properties, such as protonated ionophores at low pH, the ionized forms of the antimicrobials (pKa), affinity to divalent elements, such as Mg^++^ and Ca^++^, and solubility. We found that ethyl acetate with methanol is the most suitable extraction solvent for beta-lactams, sulfonamide, fluoroquinolone, tetracycline, macrolide antibiotics, and coccidiostats. Ethylenediaminetetraacetic acid solution was used to remove abiotic cations involved in and metallic reactions that can occur in BL. A combination of MgSO_4_ and a primary–secondary amine mixture in the QuEChER method was utilized to clean the extracted BL matrix. The MgSO_4_ reduces sorbent moisture content while the primary–secondary amine removes fatty acids and some lipids from the matrix. 

In most other studies, acetonitrile was utilized as the extraction solvent and hydrophilic-lipophilic balanced (HLB) solid phase extraction cartridges [12,27,28,29,30]. 

Methods previously described for sample preparation of broiler litter required large amounts of solvents and buffers [27,28,30,33], most of the extraction and clean-up processes utilized HLB or C-18 SPE. The SPE methods include complicated and tedious steps [33], and spent more reagent and time [28,29,30,33]. Most previous methods were specific for one or few groups of antimicrobials or coccidiostats. Compared to those methods, the analytical multi-residue method we describe includes a broad spectrum of antimicrobial and coccidiostat groups, with simple sample preparation steps with reduced use of solvents, reagents, and time.

The matrix effect was evaluated by comparing peak areas in a standard solution with the spiked extracted matrix. Signal enhancement of or suppression of different magnitude was observed in the spiked extracted matrix compared to the standard solution for beta-lactams (96%–176%), macrolides (86%–210%), tetracyclines (62%–139%), sulfonamides (85%–107%), fluoroquinolones (72%–124%), and coccidiostats (64%–76%). However, this effect was compensated by utilizing matrix-matched calibration curves for all analytes. 

The effect of composting treatment on the persistence of ionophores were also shown by Arikan [30], who found that abiotic reactions with ionophores caused a reduction in recovery, more interference, and a lower sensitivity for their detection.

The traditional in-house extraction and cleaning procedures adopted at the start of this study involved the use of an extraction buffer comprising acetone: DDW (1:4) and SPE-plexa. This resulted in low interference, good recovery, and selectivity for fluoroquinolone, tetracycline, and macrolide antimicrobials. However, this extraction method requires a large number of sample preparation steps, more solvents, and results in low recovery for other residue compounds such as beta-lactams, sulphonamides, and coccidiostats. Due to the low efficiency of pervious analytical methods, we developed a rapid multi-residue analytical method in BL to regulate the degradation and toxicity of residues in BL used as ruminant feed. Our analytical method has a lower limit of detection and a lower limit of quantification at ≤25 and ≤100 ppb, respectively. The target inter-day precision, the standard curve fit (r^2^) requirements and recoveries were <20%, ≥0.95, and 70–120% for most residue compounds in BL. This multi-residue analytical method is characterized by simple steps and a high purification effect for a large group of compounds. It also reduces the processing time, the use of organic solvents, and the use of reagents compare to previous BL analytical methods. 

Antimicrobial and coccidiostat residue concentrations varied at different locations across Israel. This might indicate differences among broiler farms using different drugs. Residue concentrations of coccidiostat, enrofloxacin, ciprofloxacin, and tetracycline in BL were low in Israel, relative to some other countries [38,39,40]. The presence of tetracycline, or fluoroquinolone groups (important antimicrobials for humans), in BL contributes to the increased rate of antibiotic-resistant pathogenic bacteria in environmental contaminations that affect human health. The low detection of sulphonamides reported here indicate a shifting away from sulphonamides to coccidiostats in poultry farming. However, the influence of coccidiostats on the development of resistant bacteria is still unclear.

Coccidiostat concentrations of maduramycin, monensin, and narasin detected before any treatment in BL were considerably below toxic concentrations in cattle and sheep [24,41,42,43,44]. During stacking treatment, the highest degradation rate was observed in the first week. This might reflect the functional activity and thus self-heating of microorganisms before termination of the fermentation process. The more biodegradable drugs were naturally produced antimicrobials as compared to synthetic compounds (logkow > 2). Our stacking treatment results showed similarity to those from past composting treatment studies on degradation of some drug residues, like tetracycline, fluoroquinolone, and some coccidiostats [45,46]. After seven days of composting treatment of turkey litter, the chlortetracycline degradation ratio was low [16], as in this study. However, there were large differences in the degradation ratios of mononsin, tylosin, and sulfamethazine (54%, 76%, and 0%, respectively) in the turkey composting study, as compared to our studies of BL degradation using a stacking treatment. Degradation of sulfadiazine by Microbacterium lacus strain SDZm4 was investigated in previous studies of soil samples [47]. Microbacterium are Gram-positive bacteria also found in broiler gut. Therefore, this may be a reason why sulfonamide degradation in broiler litter was >95% more than in turkey litter. The low degradation of erythromycin after a high concentration spike may be due to the low activity of Pseudomonas bacteria in the litter. In a previous study, *P. aerugiona* was one of bacteria that biodegraded erythromycin drugs [48]. 

In laboratory-scale study, all antimicrobials in this study were degradable <LOQ, except for tilmicosin, as seen with composting treatment for some drugs in BL [16,49,50]. However, spiked samples of tilmicosin in BL during 40 days of composting treatment at 50–60% water content were <LOQ (11 µg kg^−1^) [47]. Sun et al. explained the biodegradation of ionophores was dependent on temperature and moisture [6]. In our results, a rise in temperature with oxygen supplying led to a higher degradation ratio than seen with anaerobic treatment systems. However, the piling and turning composting system in another study assessing the degradation of salinomycin and narasin showed the opposite trend [51]. The Sun et al. studies also addressed the similarities of degradation of narasin and a synthetic of narasin with a methyl group salinomycin degradation ratio [6]. However, salinomycin degradation in our study was >95% and that of narasin was 46–58% in the stacking treatment systems. 

The treatment results with decoquinate at all three durations were unexpected. The decoquinate concentration increased with treatments. We investigate the effects of spiked concentration of a derivative of quinolone (decoquinate) and quinolones. However, the increased concentration of decoquinate were not derived from quinolones. A similar phenomenon were reported previously [52]. In that study, the stability of decoquinate and pre-mixtures (vitamin and minerals) stored at 25 °C and humidity of 60% over 6 months were 87%. However, after 18 months, the decoquinate concentration increased to 106% [52]. 

The purpose of the current study was to compare the effect of different treatments on degradation of antimicrobials and coccidiostats. Investigation of degradation product or metabolites (except for ciprofloxacin) were not included in this study. However, it would be interesting to consider this issue in a future plan.

## 4. Materials and Methods

### 4.1. Chemicals and Reagents

Erythromycin, tilmicosin, and oxytetracycline standards were purchased from Sigma-Aldrich Merck (St. Louis, MO, USA). Tylosin, decoquinate, chlortetracycline, and nicarbazin were obtained from A2S Analytical Standards Solutions (Saint Jean d’Illac, France). Ciprofloxacin, danofloxacin, norfloxacin, sulfadiazine, sulfisoxazole, and ampicillin were obtained from Toronto Research Chemicals (Toronto, ON, Canada). Enrofloxacin was obtained from Glentham (Corsham, Wiltshire, UK). Sulfadimidine, sulfachloropyridazine, sulfaquinoxaline, doxycycline, amoxicillin, maduramicin, diclazuril, and clopidol were obtained from Dr. Ehrenstorfer (Augsburg, Germany), monensin from Acros (Geel, Belgium), and lasalocid from Santa Cruz (Huissen, The Netherlands). Semduramycin came from Phibro (Teaneck, NJ, USA) and narasin from United States Pharmacopeia (Rockville, MD, USA). Robendine, salinommycin, sulfachlo-ropyrazine, and robendin-d8 were from HPC Standards (Atlanta, GA, USA). All antimicrobial and coccidiostat standards were 97–98% pure, except for nicarbazine (90%) and clopidol (95%). HPLC-grade methanol, acetonitrile, and formic acid were purchased from J. T. Baker (Deventer, The Netherlands) and EDTA was from Sigma-Aldrich Merck (Rehovot, Isrel). Ammonium formate was obtained from Fisher Chemicals (Loughborough, UK). Analytical grade ethyl acetate, acetonitrile, and methanol were obtained from Bio-Lab (Jerusalem, Israel). UV/UF-ST de-ionized water (resistivity > 18 mΩ/cm) was produced with a Thermo Scientific apparatus (Long Branch, NJ, USA).

Stock solutions containing 1000 mg kg^−1^ of the antimicrobials and coccidiostats was prepared in methanol, except for the beta-lactam solution (1000 mg L^−1^ (ppm)), which was prepared in double distilled water (DDW). Sulfonamides, fluoroquinolones, and coccidiostats were stored at 4 °C, the macrolide and tetracycline standards were stored at −20 °C and the beta-lactam standard solution was kept at −80 °C. 

### 4.2. Sample Collection

Untreated BL samples were collected from treatment companies (aerobic and stacking process-based), with 590 poultry farms providing the litter to the companies after one cycle of production. Raw litter samples (100 g) were collected from broiler farms and treatment companies at nine locations utilizing a zigzag pattern. The samples were placed into a cold container and transferred to the laboratory within 12 h, where they were frozen at −20 °C pending analysis (European Commission (EC) Regulation No. 152/2009).

### 4.3. Pre-Treatment of Raw BL

For multi-residue analytical method development, the litter was pre-treated as follows in the laboratory. Samples were freeze-dried by lyophilization and ground thoroughly to homogeneity. The moisture content of the litter at the time of collection was 30–40%. For high accuracy in multi-residue analysis (MRA), the sampling process, drying of the samples and sample homogeneity are critical. In the current study, drying the BL samples in an oven at 70 °C for 72 h degraded the majority of anti-microbials and coccidiostats (Table S4). Therefore, lyophilization was considered the preferred process for drying.

### 4.4. Sample Extraction 

**Figure 4:**
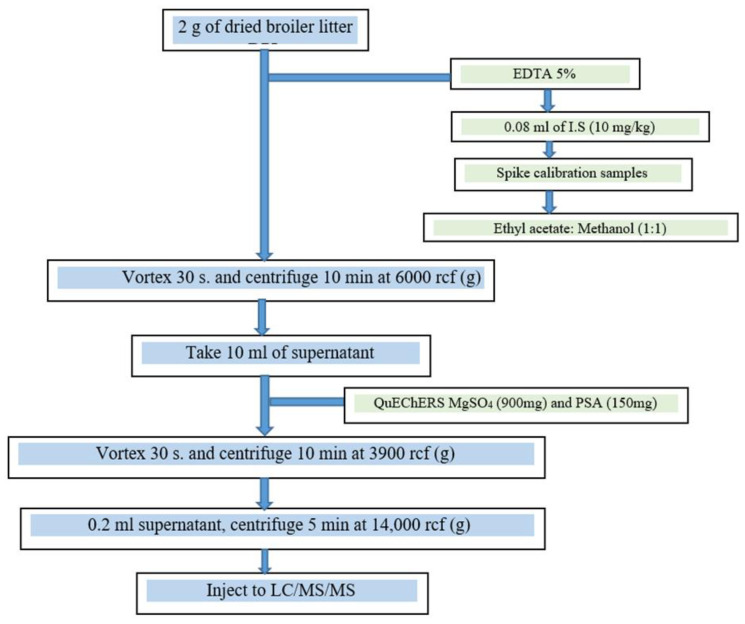
Analytical scheme for drug determination in broiler litter.

For sulfonamide, beta-lactam, tetracycline, fluoroquinolone, macrolide, and coccidiostat analysis, 10 mL ethyl acetate/methanol (1:1) and 0.08 mL of a 10 mg kg^−1^ internal standard mixture of sulfisoxazole, roxithromycin (Toronto Research Chemicals, Toronto, ON, Canada), robenidine d8 (HPLC Standards, Am Wieseneck, Germany), levofloxacin (Sigma-Aldrich, Rehovot, Israel), and minocycline (A2S Analytical Standards Solutions) were added to 2 g of BL, fortified with 30 compounds (Table S1) form the tetracycline, fluoroquinolone, sulfonamide, beta-lactam, macrolide, and coccidiostat groups at a concentration of 10 ppm each and 0.2 mL 5% EDTA, vortexed and centrifuged at 3900 rcf (*g*) for 10 min. Ten mL of the supernatant were transferred to QuEChERS (900 mg MgSO_4_, 150 mg PSA, primary and secondary amines; Restek, Bellefonte, PA, USA), vortexed and centrifuged at 3900 rcf (*g*) for 10 min, centrifuged 5 min at 14,000 rcf (*g*), and transferred 0.2 mL into a 0.25 mL vial and analyzed by LC/MS/MS. Figure 4 presents a schematic depiction of the protocol.

### 4.5. LC/MS/MS Analysis 

Antimicrobial and coccidiostat concentrations were measured using the API 4000 or 3200QTrap mass spectrometer system (Sciex, Vaughan, ON, Canada) connected to the Agilent 1200 (Agilent Technologies, Waldbronn, Germany) system (binary pump, degasser, temperature-regulated column compartment, and autosampler). Chromatographic separation of antimicrobials was achieved with a Zorbax, Eclipse Plus, C18, 4.6 mm × 50 mm, 1.8 μm (Agilent, Waldbronn, Germany) for the reversed phase column. The mobile phase consisted of 0.2% formic acid and acetonitrile. The acetonitrile gradient increased from 5% to 70% from 0 to 2 min, remained at 70% for 2 min, and then returned to the initial level at 3 min.

For coccidiostat samples, the isocratic mobile phase consisted of 10% 0.01 M ammonium formate, containing 0.1% formic acid as the aqueous solution, and 90% acetonitrile as the organic solvent, at a flow rate of 0.5 mL/min. The injection volume was 5 μL, with a post-run period of 5 min. LC/MS/MS parameters are specified in the supplementary file (Table S5). It has to be mentioned that with the 3200 instrument, samples were injected twice in positive and negative mode (not in polarity switch mode).

Adducts were observed during the optimization stage. For the majority of analytes, adducts were not chosen for monitoring, except for ionophores (lasalocid, maduramicin, monensin, narasin, and salinomycin). For these analytes, the precursor ion was the [M^+^NH4]^+^ adduct. 

Robenidine-d8 was used as an internal standard as a measure for appropriate performance of the extraction procedure, as well as for LC-MS performance, regrading proper injection and signal stability. 

### 4.6. Method Validation

The analytical method developed here was validated for recovery, accuracy, precision, suitability, specificity, and selectivity. LLOQ and LLOD were determined according to the guidelines published by the European Commission (808/2021/EC and GL49/2015) for animal products, except reproductivity and reproducibility that were 4 replications instead of 6. To the best of our knowledge, there are no published guidelines for validation with manure samples.

### 4.7. Calibration

Calibration curve and recovery: a matrix-matched calibration curve was prepared using fortified concentrations of 0, 100, 250, 500, 1000, and 1500 µg kg^−1^. Drug-free BL was spiked with a mix of antimicrobial and coccidiostat standards, extracted, cleaned, and analyzed by LC/MS/MS (Figure S1).

The expected concentration of each analyte in real samples, especially after litter treatment, was difficult to predict. Therefore, a decision was made to set the same concentration range for all analytes.

### 4.8. Recovery

Recovery of each antimicrobial and coccidiostat was calculated according to Equations (1) and (2).

Absolute recovery = PA_BL_/PA_AS_ × 100, (1)

where PA_BL_ is the peak area of the spiked BL sample, and PA_AS_ is the peak area of the clean analytical standard.

Relative recovery = SACON/CALCON × 100,(2)

where SACON is the found sample concentration and CALCON is the added sample concentration. Linear regression analysis (y = mx + b, where m = slope, b = y intercept, y = analytical peak area) was applied. Here, x = analytical spiked concentration (ppb) was used to calculate sample values.

### 4.9. Suitability (Repeatability) and Inter-Day Recovery (Reproducibility)

Injection of four replicates of known concentrations (100–1500 ppb) of fortified BL samples was performed for repeatability. Injection of four replicates of known concentrations (100–1500 ppb) of fortified broiler litter samples on four different days were analysed using two different LC/MS/MS instruments for reproducibility.

### 4.10. Specificity and Selectivity

Twenty representative blank samples and five fortified samples at the limit of quantitation were assessed by LC/MS/MS to determine the specificity and selectivity of each compound.

### 4.11. Matrix Effect

Matrix effect was evaluated by comparing peak area of standard solution with spiked extracted matrix. 

### 4.12. Designing a Method for Stacking BL Treatment Processes in the Laboratory

Broiler litter was removed from the facility over the course of a year. Stacking treatments were carried out in three separate jars containing 12 kg of BL over the course of three weeks. The initial moisture content balanced at 40%. The degradation rate for the stacking treatment was determined every week. Broiler litter free of drugs that originated from an antibiotic independent farm was spiked at initial concentrations of 5 mg kg^−1^ (low level), 10 mg kg^−1^ (medium level), 15, and 20 mg kg^−1^ (high level) with a mix of five representative antimicrobial drugs, namely, sulfisoxazole, amoxicillin, erythromycin, tetracycline, and ciprofloxacin. A mix of 29 antimicrobials used in poultry farming and coccidiostats was also spiked at low concentrations of 0.5 mg kg^−1^ (low level), 1 mg kg^−1^ (medium level), 1.5, and 2 mg kg^−1^ (high level). Spiked BL samples were wrapped in cotton gauze fabric and mixed with BL in jars before each treatment and placed at the medium (*n* = 4) and upper layers (*n* = 4).

## 5. Conclusions

This study described a rapid method for simultaneous detection of a large number of antimicrobials and coccidiostats at residue concentrations. Effective extraction, cleaning, identification and quantification methods were essential to achieve good recovery and repeatability in a large number of samples containing low levels of pharmaceutical compounds in a complex matrix. The multi-residue analytical method reduced a large number of sample preparation steps and reduced the high analytical cost compared with traditional methods. This method offers an affordable tool for monitoring and understanding the presence, persistence, degradation, and relation between residue drugs and resistant bacteria in BL. Thus, this method will help to understand and mitigate animal and human health problems, as well as environmental effects, caused by antimicrobial and coccidiostat drug residues. The use of antimicrobials and coccidiostats in animal farming is of paramount concern with respect to human, animal, and environmental contamination issues and as a source of pathogenic-resistant bacteria. Still, most farmers use BL as ruminant feed and fertilizer without processing. Even though the effects of stacking treatment on degradation of antimicrobials were significantly high, the degradation of coccidiostats and tilmicosin were limited. At the same time, further study on decoquinate concentration-increasing phenomenon during treatments is needed. 

This study supports the need for better understanding, regulation, management, and proper use of animal waste so as to protect and reduce the numbers of antibiotic-resistant bacteria and use of antimicrobials that carry a high risk for animals, humans, and the environment.

## Figures and Tables

**Table 1:** Intra-day analytical recovery (in %) of 30 antimicrobials and coccidiostats in broiler litter.

Group	Analyte	0.1 mg kg^−1^	RSD %	1.5 mg kg^−1^	RSD %
Coccidiostats	Narasin	76	10	92	20
	Diclazuril	95	4	96	5
	Clopidol	101	9	97	7
	Nicarbazine	80	20	95	4
	Maduramycin	88	6	84	11
	Monensin	87	11	96	4
	Robenidine	116	10	105	2
	Salinomycin	70	12	95	4
	Semduramycin	112	11	101	9
	Lasalocid	113	6	101	0
	Decoquinate	105	9	105	2
Sulfonamides	Sulfachloropyrazine	101	20	102	5
	Sulfachloropyridazine	110	8	101	2
	Sulfadiazine	112	17	109	2
	Sulfadimidine	130	15	99	12
	Sulfaquinoxaline	87	4	98	5
	Sulfadoxine	124	7	107	2
Macrolides	Tilmicosin	103	12	104	2
	Tylosin	84	12	94	1
	Erythromycin	116	19	104	8
Fluoroquinolones	Danofloxacin	124	15	103	2
	Ciprofloxacin	128	17	105	0
	Norfloxacin	85	19	101	4
	Enrofloxacin	114	17	103	2
Tetracycline	Doxycycline Hyclate	100	14	111	7
	Oxytetracycline	102	5	97	24
	Chlortetracycline	116	19	104	8
	Tetracycline	84	5	102	4
b-Lactams	Amoxicillin trihydrate	102	14	112	18
	Ampicillin	96	10	102	5

## Data Availability

Data will be available on request from S.J.M.

## Supplementary Materials

The following supporting information can be downloaded at: https://www.mdpi.com/article/10.3390/ph17020203/s1, Table S1: Antibiotics and coccidiostats approved for use in poultry by national regulatory authorities in the USA, Brazil, China, Poland, United Kingdom, Germany, France, Israel, and Spain, based on national reports as described in Section 1; Table S2: Intra-day analytical recovery (in %) of 30 anti-microbials and coccidiostats in poultry litter as described in Section 2.1.1; Table S3: Inter-day analytical recovery (in %) of 30 antimicrobials and coccidiostats in broiler litter as described in Section 2.1.2; Table S4: Comparison of antimicrobial and coccidiostat degradation in poultry litter upon drying by oven or lyophilzation described in Section 4.2; Table S5: LC/MS/MS conditions as described in Section 4.2; Table S6: Determination of signal to noise (S/N) at the LOQ levels; Figure S1: Calibration curves for some antimicrobials and coccidiostats in fortified PL (*n* = 5) as described in Section 4.7; Figure S2: Chromtograms of beta-lactams in standard solution (a), blank matrix (b), and spiked matrix (c), Figure S3: Chromatograms of coccidiostats (positive mode) in standard solution (a), blank matrix (b), and spiked matrix (c); Figure S4: Chromatograms of coccidiostats (negative mode), in standard solution (a), blank matrix (b), and spiked matrix (c).
