# Supplementary material for: A Multi-Residue Analytical Method for Assessing the Effects of Stacking Treatment on Antimicrobial and Coccidiostat Degradation in Broiler Litter
Source: Pharmaceuticals (Basel). 2024 Feb 4;17(2):203. doi: 10.3390/ph17020203 (PMC10892054; doi:10.3390/ph17020203)
Supplement: Supplementary file 1 [file pharmaceuticals-17-00203-s001.zip › pharmaceuticals-2754995-supplementary.pdf]

Supplementary information

A Multi-Residue Analytical Method for Assessing the Effects of Stacking Treatment on Antimicrobial and Coccidiostat Degradation in Broiler Litter

Solomon Efriem [1,2], Malka Britzi [2], Stefan Soback [2], Chris Sabastian [1] and Sameer J. Mabjeesh [1,*]

[1] The Robert H. Smith Faculty of Agriculture, Food and Environment, Hebrew University, P.O. Box 12, Rehovot 7610001, Israel
[2] National Residue Control Laboratory, Kimron Veterinary Institute, Beit Dagan 5025001, Israel
* Correspondence: sameer.mabjeesh@mail.huji.ac.il; Tel.: +972-8-9489314

Table S1. Antibiotics and coccidiosats approved for use in poultry by national regulatory authorities in the USA, Brazil, China, Poland, United Kingdom, Germany, France, Israel and Spain, based on national reports as described in Section 1.

Coccidiostates	Fluoroquinolones	beta-Lactams	Tetracyclines	Macrolides	Sulfonamides
Lasalocid	Ciprofloxacin**	Amoxicillin	Chlortertacycline	Tilmicosin	Sulfachloropyrazine
Maduramicin	Danofloxacin	Ampicillin	Doxycycline	Tylosin	Sulfachloropyridazine
Monensin	Enrofloxacin		Oxytetracycline	Erythromycin*	Sulfadiazine
Narasin	Norfloxacin		Tetracycline		Sulfadimethoxine
Salinomycin					Sulfisoxazole*
Semduramycin					Sulfadimidine
Diclazuril					
Robenidine					
Decoquinate					
Clopidol					
Nicarbazine					

Source, Israeli Drug Registry [27].

* Not approved in Israel

** Metabolite of enrofloxacin and used in humans

Table S2. Intra-day analytical recovery (in %) of 30 anti-microbials and coccidiostats in poultry litter as described in Section 2.1.1.

Group	Analyte	0.1 mg•kg^{-1}	0.5 ppm mg•kg^{-1}	1.5 ppm mg•kg^{-1}	RSD %
Coccidiostats	Narasin	76	86	92	4
	Diclazuril	95	113	96	10
	Clopidol	101	103	97	3
	Nicrbazine	80	98	95	10
	Maduramicin	88	90	84	3
	Monensin	87	96	96	5
	Robenidine	116	111	105	6
	Salinomycin	70	90	95	13
	Semduramycin	112	115	101	7
	Lasalocid	113	113	101	7
	Decoquinate	105	80	105	14
Sulfonamides	Sulfachloropyrazine	101	102	102	1
	Sulfachloropyridazine	110	98	101	6
	Sulfadiazine	112	117	109	4
	Sulfadimidine	130	112	99	16
	Sulfaquinoxaline	87	96	98	6
	Sulfadoxine	124	117	107	9
Macrolides	Tilmicosin	103	111	104	4
	Tylosin	84	94	94	6
	Erythromycin	116	97	104	10
Fluoroquinolones	Danofloxacin	124	106	103	11
	Ciproflaxacin	128	105	105	13
	Norfloxacin	85	97	101	8
	Enrofloxacin	114	119	103	8
Tetracyclines	Doxycycline hyclate	100	102	111	6
	Oxytetracycline	102	95	97	4
	Chlortetracycline	116	110	104	6
	Tetracycline	84	118	102	17
b-Lactams	Amoxicillin trihydrate	102	94	112	9
	Ampicillin	96	104	102	4

Table S3. Inter-day analytical recovery (in %) of 30 antimicrobials and coccidiostats in broiler litter as described in Section 2.1.2.

Group	Analyte	**0.1** mg•kg^{-1}	**0.5** mg•kg^{-1}	**1.5** mg•kg^{-1}	RSD %
Coccidiostats	Narasin	67	102	99	20
	Diclazuril	83	99	96	9
	Clopidol	74	110	103	19
	Nicrbazine	80	101	98	12
	Maduramicin	89	107	100	9
	Monensin	96	101	99	3
	Robenidine	76	104	99	15
	Salinomycin	84	84	100	9
	Semduramycin	89	98	102	6.7
	Lasalocid	92	97	99	3.6
	Decoquinate	94	109	97	8
Sulfonamides	Sulfachloropyrazine	130	105	99	16.5
	Sulfachloropyridazine	83	101	99	10
	Sulfadiazine	105	101	100	2.6
	Sulfadimidine	86	105	105	11
	Sulfaquinoxaline	91	98	99	4.4
	Sulfadoxine	74	93	115	20
Macrolides	Tilmicosin	70	100	98	20
	Tylosin	76	100	96	12
	Erythromycin	112	107	97	7
Fluoroquinolones	Danofloxacin	68	102	102	5
	Ciproflaxacin	94	101	104	5.2
	Norfloxacin	68	93	110	25
	Enrofloxacin	86	104	101	9.6
Tetracyclines	Doxycycline Hyclate	70	112	98	17
	Oxytetracycline	124	124	103	12
	Chlortetracycline	70	116	96	23
	Tetracycline	78	115	99	19
b-Lactams	Amoxicillin trihydrate	103	108	107	2.5
	Ampicillin	101	87	103	8.5

Table S4. Comparison of antimicrobial and coccidiostat degradation in poultry litter upon drying by oven or lyophilzation described in Section 4.2.

Compound	Oven to Lyophilisation ratio	Control to Lyophilisation ratio	Compound	Oven to Lyophilisation ratio	Control to Lyophilisation ratio
Sulfachloropyrazine	0.28	0.95	Monensin	0.54	0.76
Sulfadimidine	0.58	0.96	Salinomycin	0.43	0.9
Sulfadiazine	0.11	0.93	Semduramycin	0.42	0.9
Sulfadoxine	0.57	0.78	Lasalocid	0.49	0.95
Sulfadimethoxine	0.53	1.1	Narasin	0.4	0.82
Sulfaquinoxaline	0.85	0.9	Decoquinate	0.34	0.57

Compound	Oven to Lyophilisation ratio	Control to Lyophilisation ratio	Compound	Oven to Lyophilisation ratio	Control to Lyophilisation ratio
Sulfachloropyridazine	0.43	1	Robenidine	0.5	0.74
Doxycycilin	0.07	0.95	Maduramycin	0.51	0.7
Oxytetracycline	0.53	0.62	Nicarbazine	0.75	0.9
Chlortetracycline	0.47	1.03	Enrofloxacin	0.71	0.89

Table S5. LC/MS/MS conditions as described in Section 4.2.

Antimicrobials

Analytical column: Zorbax, Eclipse Plus, C18, 4.6×50 mm, 1.8μ, Agilent.
Mobile phase: A: Formic acid 0.2% B: acetonitrile
Volume of injection: 3 μl
Column oven: 40°C

Gradient conditions:

	Total Time (min)	Flow rate (μl/min)	A %	B %
1	0	1200	95	5
2	1	1200	30	70
3	1.5	1200	30	70
4	1.7	1200	95	5
5	3	1200	95	5

MS/MS parameters

Source: ESI
Polarity: positive
Scan type: MRM
Source:
CAD: 6
CUR: 35
IS: 5500
TEM: 600
GS1: 80
GS2: 60
Dwell time: 5msec

Q1	Q3	ID	DP (volts)	EP (volts)	CE (volts)	CXP (volts)	R.T
Fluoroquinolones							

Q1	Q3	ID	DP (volts)	EP (volts)	CE (volts)	CXP (volts)	R.T
360.2	316.4	Enrofloxacin.1	86	10	29	0	1.79
360.2	245.2	Enrofloxacin.2	86	10	37	6	1.79
332	314	Ciprofloxacin.1	91	10	33	8	1.76
332	231	Ciprofloxacin.2	86	10	33	8	1.76
358	340	Danofloxacin.1	56	10	31	10	1.78
358	255	Danofloxacin.2	56	10	31	10	1.78
320	302	Norfloxacin.1	86	10	31	10	1.75
320	231	Norfloxacin.2	81	10	31	10	1.75
Sulfonamides							
285	156	Sulfachloropyridazine.1	51	10	31	28	1.98
285	92	Sulfachloropyridazine.2	51	10	31	28	1.98
254	156	Sulfamethoxazole.1	66	10	23	28	2.01
254	92	Sulfamethoxazole.2	66	10	23	28	2.01
251	156	Sulfadiazine.1	66	10	23	28	1.74
251	92	Sulfadiazine.2	66	10	23	28	1.74
256	156	Sulfathiazole.1	66	10	23	28	1.77
256	92	Sulfathiazole.2	61	10	23	28	1.77
279	186	Sulfadimidine.1	71	10	25	34	1.91
279	124	Sulfadimidine.2	66	10	25	34	1.91
311	156	Sulfadoxine.1	71	10	29	28	2.01
311	92	Sulfadoxine.2	81	10	29	28	2.01
301	156	Sulfaquinoxaline.1	81	10	25	28	2.08
301	92	Sulfaquinoxaline.2	76	10	25	28	2.08
268	92	Sulfisoxazole.1	66	10	37	8	2.03
268	113	Sulfisoxazole.2	66	10	37	8	2.03
Tetracyclines							
479	444	Chlortetracycline.1	76	10	31	12	1.87
479	462	Chlortetracycline.2	71	10	31	12	1.87
445	428	Doxycycline.1	76	10	29	12	1. 89
445	267	Doxycycline.2	71	10	29	12	1.89
461	426.2	Oxytetracycline.1	81	10	27	12	1.76
461	443.1	Oxytetraciclyne.2	81	10	19	12	1.76
445.1	410.14	Tetracycline.1	61	10	27	6	1.8
445.1	427.1	Tetracycline.2	61	10	19	12	1.8
Macrolides							
734.3	576.4	Erythromycin.1	61	10	27	18	1.95
734.3	558.4	Erythromycin.2	61	10	27	16	1.95
869.6	696.5	Tilmicosin.1	171	10	51	22	1.86
869.6	522.6	Tilmicosin.2	171	10	61	18	1.86
916.6	772.5	Tylosin.1	116	10	39	22	1.97
916.6	598.5	Tylosin.2	116	10	45	20	1.97
806.7	420.5	Tulathromycin.2	111	10	45	14	1.72
Beta-lactams							
366.2	349.1	Amoxicillin.1	61	10	13	10	1.82

Q1	Q3	ID	DP (volts)	EP (volts)	CE (volts)	CXP (volts)	R.T
366.2	114	Amoxicillin.2	61	10	27	10	1.82
350.1	106.1	Ampicillin.1	56	10	29	8	1.86
350	114	Ampicillin.2	71	10	31	10	11.860

Coccidiostats

Analytical column: Synergi fusion 4μm, 2×50 mm, Phenomenex
Mobile phase: Channel A: Ammonium formate 0.01M pH +0.1% formic acid

Channel B: Acetonitrile
Mobile phase time table:

time (min)	flow rate (μL/min)	channel A	channel B
0	600	90	10
5	600	10	90
6.5	600	10	90
6.7	600	90	10
9.5	600	90	10

Volume of injection: 5μl
Column oven: 40°C
Source: ESI
Scan type: MRM
Source:
CUR: 20
IS: -4500
TEM: 550
GS1: 45
GS2: 45

MS parameters:

Q1 (Da)	Q3 (Da)	Time (msec)	ID	DP (v)	EP(v)	CE (v)	CEP (v)	CXP (v)	R.T
Positive polarity									
934.6	629.4	20	Maduramicin.1	51	4.5	70	37	6	6.86
934.6	393.3	20	Maduramicin.2	51	4.5	70	39	6	6.86
688.4	461.3	20	Monensin.1	36	4.5	80	33	6	8.16
688.4	635.5	20	Monensin.2	36	4.5	80	25	6	8.16
768.5	733.6	20	Salinomycin.1	46	4.5	36	25	6	8.17
768.5	373.4	20	Salinomycin.2	46	4.5	36	41	6	8.17
608.3	237.3	20	Lasalocid.1	21	6.5	24	31	4	7.31
608.3	337.4	20	Laslocid.2	21	6.5	24	25	4	7.31
782.3	747.4	20	Narasin.1	56	6.5	32	33	8	6.68
782.3	373.2	20	Narasin.2	56	6.5	32	43	6	6.69

418.2	372.2	20	Decoquinate.1	56	4.5	18	29	6	6.4
418.2	204.1	20	Decoquinate.2	56	4.5	18	55	6	6.4
334.1	137.9	20	Robenidine.1	51	9	18	33	4	4.38
334.1	155.2	20	Robenidine.2	51	9	18	27	4	4.38
416.1	100.2	20	Hlofuginone.1	51	5	22	37	4	3.35
416.1	120.1	20	Hlofuginone.2	51	5	22	27	4	5.29
192	101	20	Clopidol.1	71	11	14	35	4	1.26
192	128	20	Clopidol.2	71	10.5	14	35	4	1.22
342.1	142.1	20	Robenidine-d8.1 (i.std)	56	8.5	16	33	4	
Negative polarity									
301	137	-30	Nicabazin.1	-9.5	-14	-20	-2	301	5.06
301	107	-30	Nicarbazin.2	-10	-14	-20	-2	301	5.07
405	334	-45	diclazuril.1	-4.5	-18	-20	-4	405	5.4
407	336	-55	diclazuril.2	-4	-16	-20	-4	407	5.4

TableS6. Determination of signal to noise (S/N) at the LOQ levels.

		Peak Area Standard (0.1 mg•kg-1)	CV%	Peak Area LOQ (0.1 mg•kg-1)	CV%	Peak Area Blank Samples	CV%	S/N
Coccidiostats	Narasin	179333.3	7.6	128666.7	17.2	2613.8	11.5	49
	Clopidol	66033.3	12.6	23500.0	2.0	N.D	N.D	>10*
	Nicarbazine	231333.3	7.0	308000.0	2.5	5410.0	6.3	56.9
	Maduramycin	48200.0	3.3	36433.3	1.7	N.D	N.D	>10*
	Monensin	68666.7	1.8	108333.3	2.8	6430.9	5.2	16.8
	Robenidine	93700.0	1.4	22933.3	2.4	1475.7	2.9	15.5
	Salinomycin	193333.3	6.5	196333.3	1.1	5076.9	3.9	38.7
	Semduramycin	15200.0	0.7	47200.0	5.0	N.D	N.D	>10*
	Lasalocid	128333.3	1.6	65600.0	0.7	971.3	4.2	67.5
	Decoquinate	918000.0	3.9	355000.0	3.7	46672.1	0.7	**7.6**
Sulfonamides	Sulfachloropyrazine	42600.0	7.4	21333.3	15.5	N.D	N.D	>10*
	Sulfachloropyridazine	155333.3	14.2	63366.7	13.8	5705.1	20.7	11.1
	Sulfadiazine	657000.0	1.8	330000.0	4.8	N.D	N.D	>10*
	Sulfadimidine	437666.7	0.7	225666.7	6.7	N.D	N.D	>10*
	Sulfaquinoxaline	346333.3	10.9	156666.7	2.1	N.D	N.D	>10*
Macrolides	Tilmicosin	24400.0	5.4	1613.3	2.9	N.D	N.D	>10*
	Tylosin	576000.0	4.7	1426.7	11.0	N.D	N.D	>10*
	Erythromycin	10766.7	6.8	416.7	21.8	N.D	N.D	>10*
Fluoroquinolones	Danofloxacin	1533333.3	0.8	95900.0	5.4	N.D	N.D	>10*
	Ciprofloxacin	725000.0	4.1	25933.3	2.6	N.D	N.D	>10*
	Norfloxacin	817666.7	6.6	34866.7	3.2	N.D	N.D	>10*
	Enrofloxacin	1546666.7	7.1	91300.0	1.3	N.D	N.D	>10*
Tetracycline	Doxycycline	184666.7	1.6	333000.0	4.4	N.D	N.D	>10*
	Oxytetracycline	143333.3	4.3	165666.7	1.5	N.D	N.D	>10*
	Chlortetracycline	13733.3	0.1	332000.0	8.5	N.D	N.D	>10*
	Tetracycline	227333.3	7.1	375333.3	1.8	N.D	N.D	>10*
beta-Lactams	Amoxicillin	21366.7	7.5	12433.3	6.8	N.D	N.D	>10*
	Ampicillin	74166.7	5.9	48466.7	3.8	N.D	N.D	>10*

N.D; Not detected; * no peak detected in blank.

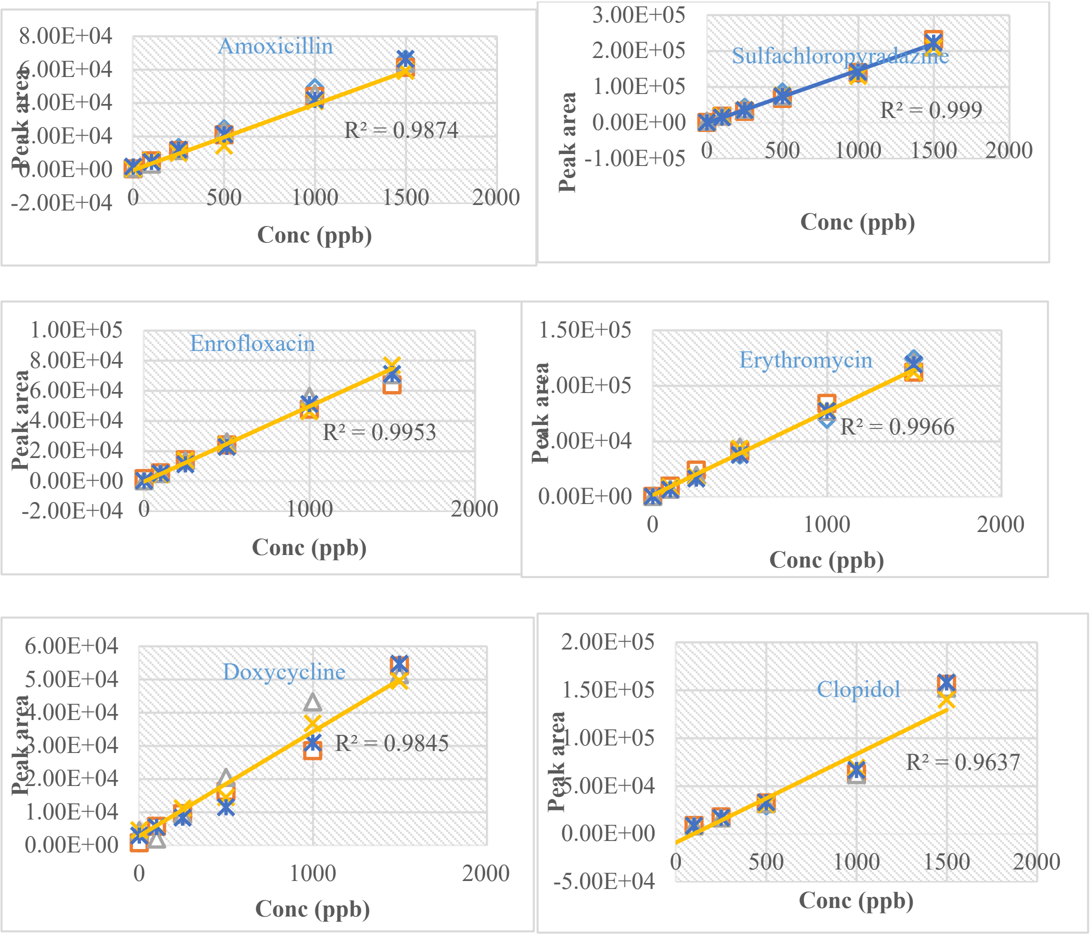

Figure S1. Calibration curves for some antimicrobials and coccidiostats in fortified PL (n = 5) as described in Section 4.7.

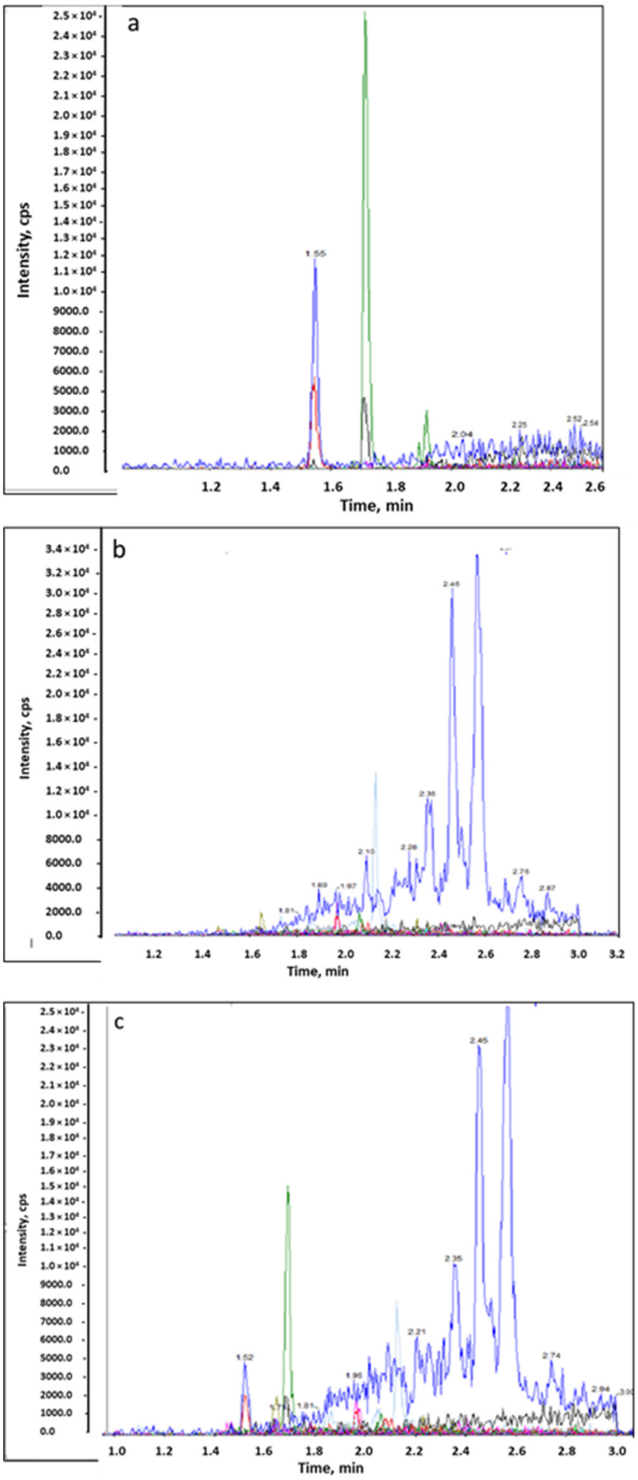

Figure S2. Chromtograms of beta-lactams in standard solution (a), blank matrix (b), and spiked matrix (c).

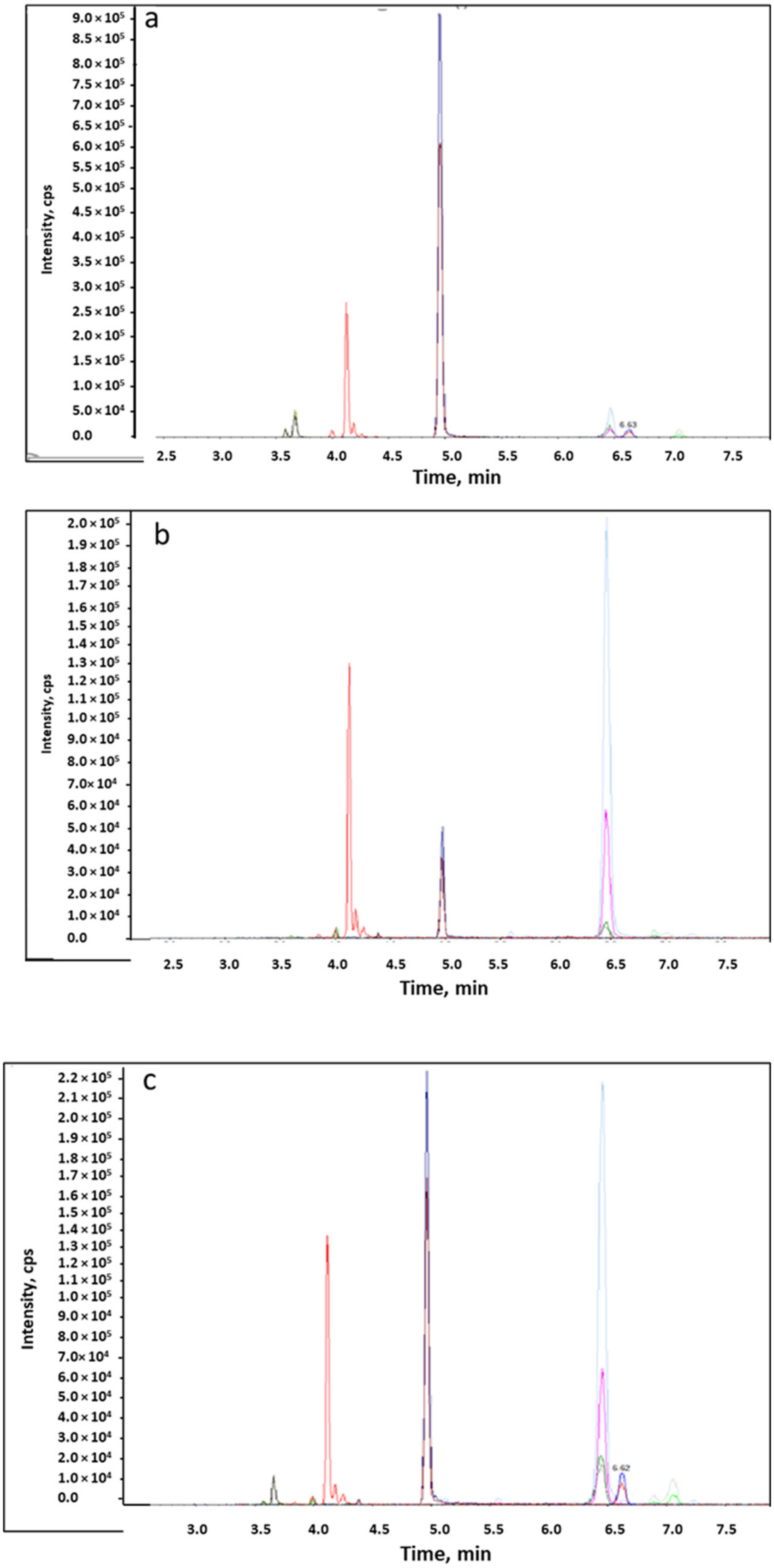

Figure S3. Chromtograms of coccidiostats in positive mode, standard solution (a), blank matrix (b), and spiked matrix (c).

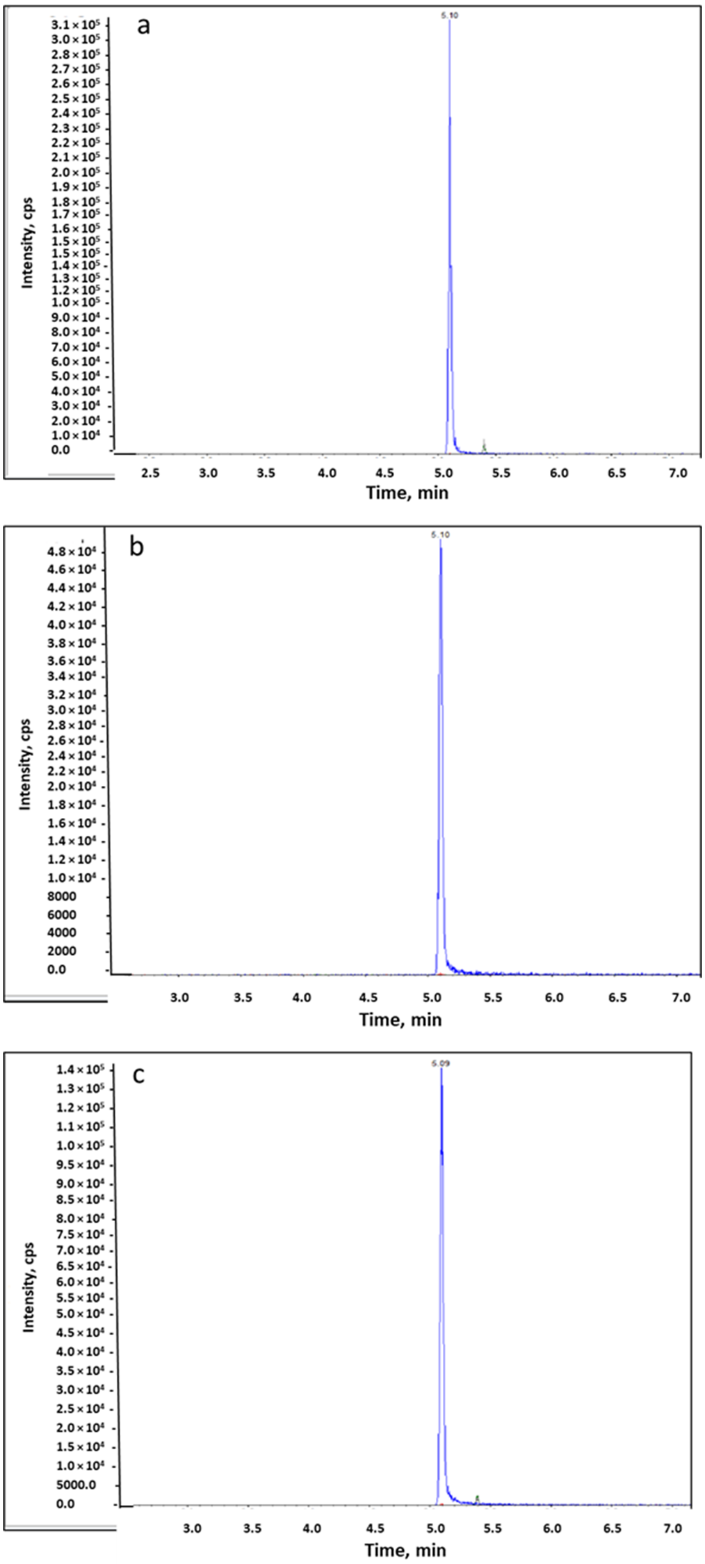

Figure S4. Chromtograms of coccidiostats in negative mode, standard solution (a), blank matrix (b), and spiked matrix (c).
